# Strong Coupling and Nonextensive Thermodynamics

**DOI:** 10.3390/e22090975

**Published:** 2020-09-01

**Authors:** Rodrigo de Miguel, J. Miguel Rubí

**Affiliations:** 1 Department of Teacher Education, Norwegian University of Science and Technology, 7491 Trondheim, Norway; 2 Department of Condensed Matter Physics, University of Barcelona, 08007 Barcelona, Spain; mrubi@ub.edu; 3 PoreLab—Center of Excellence, Norwegian University of Science and Technology, 7491 Trondheim, Norway

**Keywords:** thermodynamics at strong coupling, nonextensive thermodynamics, thermodynamics of small systems, *temperature-dependent* energy levels, interfacial properties

## Abstract

We propose a Hamiltonian-based approach to the nonextensive thermodynamics of small systems, where *small* is a relative term comparing the size of the system to the size of the effective interaction region around it. We show that the effective Hamiltonian approach gives easy accessibility to the thermodynamic properties of systems strongly coupled to their surroundings. The theory does not rely on the classical concept of *dividing surface* to characterize the system’s interaction with the environment. Instead, it defines an effective interaction region over which a system exchanges extensive quantities with its surroundings, easily producing laws recently shown to be valid at the nanoscale.

## 1. Introduction: The System and Its Surroundings

Systems are never truly isolated. They are in contact with an environment which influences their energy, volume, and mass. In certain cases, the presence of the environment is of little significance for the properties of the system, and, for simplicity, the system may be described as if it were isolated. In other cases, surroundings significantly affect the properties of systems, and external interactions need to be taken into account. Systems subject to the latter scenario are *nonextensive*, meaning that their extensive properties do not scale linearly with one another [1].

Small-sized systems, such as single molecules [2], atomic clusters [3], biopolymers [4], molecular motors [5], or nanoporous membranes [6,7], are typical examples of nonextensive systems. The energy, mass, and volume of these systems can be significantly altered by what is around them, and their scale related properties often escape the paradigms of classical thermodynamics [8]. Small-sized systems may even exhibit anomalous properties such as negative heat capacity [9] and thermophilic motion [10]. In contrast to small systems, the properties of their macroscopic analogs usually exhibit negligible variations from their mean isolated values.

Yet, a system need not be small in size in order to be nonextensive. Macroscopic systems may also exhibit this property, for what determines whether or not a system is *small* is not its sheer size, but how the size compares to the range of the interactions affecting the system [1,11].

**Figure 1:**
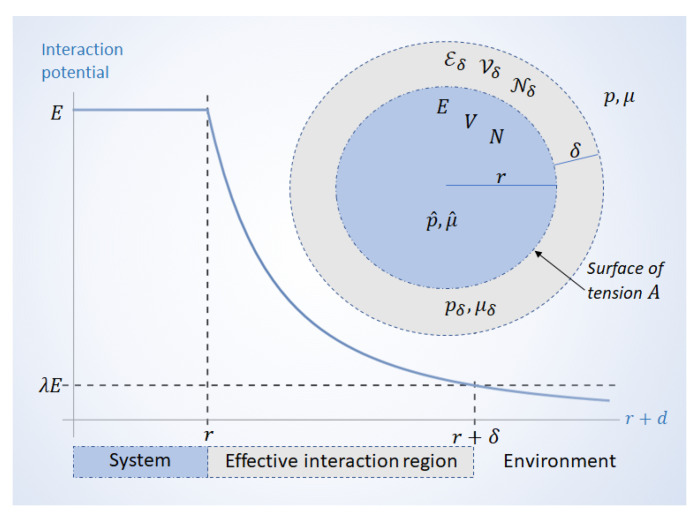
*Graph*: Interaction potential of a spherical system coupled to its environment. The potential decays as ${\left(r+\delta \right)}^{-\alpha }$. The effective interaction region extends over a distance δ which fulfills condition (1). *Diagram*: The system has energy *E*, volume *V*, and *N* particles. The system’s pressure $\hat{p}$ and chemical potential $\hat{\mu }$ differ from the environment by an amount $p_{\delta }=\hat{p}-p$, and $\mu_{\delta }=\hat{\mu }-\mu$, where $p_{\delta }$ and $\mu_{\delta }$ are, respectively, the pressure and chemical potential at the effective interaction region. The interaction region is a phase with thickness δ, volume $V_{\delta }$, energy $E_{\delta }$, and $N_{\delta }$ particles.

To see this, we may consider a spherical system with radius *r*. There is an effective interaction region around the system; how far this region extends beyond the surface depends on how quickly the interaction potential decays as a function of distance. This is illustrated in Figure 1. If the interaction potential around the system decays as as a function of the distance *d* from the surface as ${\left(r+d\right)}^{-\alpha }$ (for some positive α), then the effective interaction region may be defined by the largest distance δ that fulfills the condition

(1) $${\left(r+\delta \right)}^{-\alpha }>\lambda \;r^{-\alpha }$$

for some λ∈(0,1). The parameter λ is the tolerance of the approximation that is necessarily made in describing the system as separate from its environment; together with α (the exponent that describes how interactions fade out) it determines when the inclusion of the environment $\left(+\delta \right)$ stops being significant. A smaller tolerance λ would demand a greater δ.

Once the effective interaction region is included, the total volume V is given by

(2) $$V=V+V_{\delta }$$

where $V\equiv 4\pi r^{3}/3$ is the volume of the bare system, and $V_{\delta }\equiv 4\pi {\left(r+\delta \right)}^{3}/3-V$ is the volume of the interaction phase surrounding it. Likewise, the total energy E and number of particles N are given by

(3) $$E=E+E_{\delta },$$

(4) $$N=N+N_{\delta },$$

where *E* and *N* are, respectively, the energy and number of particles in the absence of interactions, and $E_{\delta }$ and $N_{\delta }$ are the energy and amount of particles in the interaction region, respectively.

When the thickness δ of the effective interaction region is small, the resulting perturbations are small, and they only become significant for small *r* systems. However, if the interaction region is large, i.e., large δ, then perturbations can be significant also for large *r* systems. It is the parameter α that determines how the size of the interaction region compares to the system. Their length scales are related by

(5) $$\frac{r+\delta }{r}<\lambda^{-1/\alpha },$$

and their volumes by

(6) $$\frac{V}{V}<\lambda^{-3/\alpha }.$$

When α is large, the interaction region does not reach very far, and the system interacts with its environment only through a relatively thin boundary. On the other hand, when α is small, δ may far exceed the system’s radius, making it capable of significant interactions with systems that are *far* away. In either case, when the interactions are significant, the system’s energy is no longer extensive, and it needs to be described by an effective Hamiltonian that accounts for perturbations from the environment.

In this work, we show that a thermostatitical framework based on an effective *Hamiltonian of mean force* yields a theory very well suited to describing the thermodynamic properties of nonextensive systems. The Hamiltonian framework does not rely on the concept of surface to characterize the system’s interaction with the environment. Instead, it defines an effective interaction region over which a system exchanges extensive quantities with its surroundings. This provides insight into interphases beyond the *dividing surface of discontinuity* paradigm, and it predicts thermodynamic relations valid at the nanoscale.

The rest of the paper is organized as follows. In Section 2, a Hamiltonian of mean force including strong interactions with the environment is briefly introduced. In Section 3, we use the Hamiltonian of mean force to describe how the effective thermodynamic properties of the strongly coupled system deviate from those of the bare system. Section 4 focuses on the interaction region surrounding the system—the interphase, producing thermodynamic laws valid at the nanoscale in a rather simple manner. Final remarks are given in Section 5.

## 2. Hamiltonian of Mean Force: A Framework for Nonextensive Thermodynamics

The Hamiltonian of mean force is an extended Hamiltonian that accounts for interactions with the environment. It forms the basis of thermodynamics at strong coupling [12,13,14], a framework recently shown to provide a simple thermostatistical description of negative thermophoresis [15].

If a system A in state *a* can exchange energy with a bath in state η, then the total energy is given by the sum of the system’s energy $H_{a}^{A}$, the bath’s energy $H_{\eta }^{\mathrm{BATH}}$, and the interaction energy $I_{a,\eta }^{A,\mathrm{BATH}}$ between the system and the bath. Averaging the sum of the bare system’s energy and the interaction energy over the bath results in a Hamiltonian of mean force $E_{a}^{A}$ for the system given by

(7) $$e^{-\beta E_{a}^{A}}=\frac{\sum_{\eta }e^{-\beta \left(H_{a}^{A}+I_{a,\eta }^{A,\mathrm{BATH}}+H_{\eta }^{\mathrm{BATH}}\right)}}{\sum_{\eta }e^{-\beta H_{\eta }^{\mathrm{BATH}}}},$$

where $\beta \equiv 1/k_{B}T$, $k_{B}$ is Boltzmann’s constant, and *T* is the temperature.

If the coupling energy term $I_{a,\eta }^{A,\mathrm{BATH}}$ is negligible compared to the system’s own energy $H_{a}^{A}$, then the effective Hamiltonian simply reduces to the bare system’s Hamiltonian $H_{a}^{A}$. However, for systems with sufficiently low energy or sufficiently strong interactions, the system’s energy is comparable to the interaction energy, and the latter may no longer be neglected. Indeed, the presence of the interaction $I_{a,\eta }^{A,\mathrm{BATH}}$ causes the effective Hamiltonian $E_{a}^{A}$ to be temperature-dependent, as long suggested by Elcock and Landsberg [16] and others [15,17,18,19,20].

As the Hamiltonian of mean force captures the effects of the interaction region surrounding a system, it is a natural starting point towards a thermodynamic description of systems, large or small, whose scale related properties break the paradigms of extensive (i.e., classical) thermodynamics.

Meanwhile, it must be noted that the Hamiltonian approach of thermodynamics at strong coupling (TSC) is fundamentally different from the classical thermodynamic framework proposed by Hill in the early 1960s, where it was proposed that Euler’s equation (i.e., extensivity) be corrected by fictitiously replicating a system and then regarding it as a member in a homogeneous collection of many identical subsystems interacting with a so-called replica energy [21,22]. Instead, TSC takes into account the microscopic origin of nonextensivity from the outset, offering a framework well suited to the modeling and simulation of complex systems (large or small) subject to strong interactions.

## 3. Strongly Coupled System

If we consider a closed system with volume V and N particles in contact with a bath at inverse temperature β, the partition function Z may be written using the effective energy (7):

(8) $$Z=\sum_{i}e^{-\beta E_{i}^{A}},$$

where, due to the nonvanishing interaction term in (7), $E_{i}^{A}$ is temperature-dependent. In the following, and for notational simplicity, we drop the superindex *A* used to label the system in Equation (7). The partition function may be used to find the internal energy E of the system:

(9) $$E=-\frac{\partial }{\partial \beta }\log Z,$$

resulting in expression (3) with

(10) $$E\equiv \langle E_{i}\rangle ,$$

and

(11) $$E_{\delta }\equiv -T\langle \frac{\partial E_{i}}{\partial T}\rangle ,$$

where $\langle \cdot \rangle$ denotes the average over all microstates of the bare system.

Expression (3) gives us the effective internal energy. The quantity *E* is the reference energy for the bare system in the absence of coupling. The additional term $E_{\delta }$ is an excess energy resulting from the strong interactions happening at the effective interaction region; when those interactions are absent (isolated system) or negligible (extensive system), Equation (7) simplifies and the derivative in (11) is simply zero.

The system’s effective pressure $\hat{p}$ may be defined as

(12) $$\hat{p}=-\frac{\partial E}{\partial V},$$

with E given by (3). We may then write

(13) $$\hat{p}=p+\Delta p,$$

where p≡−∂E/∂V is the pressure in the absence of coupling, and

(14) $$\Delta p\equiv -\frac{\partial E_{\delta }}{\partial V}$$

is the additional pressure due to the energy exchange with the environment through the interaction region.

Likewise, the effective chemical potential $\hat{\mu }$ may be defined as

(15) $$\hat{\mu }=\frac{\partial E}{\partial N},$$

which, invoking (3), becomes

(16) $$\hat{\mu }=\mu +\Delta \mu ,$$

where μ≡∂E/∂N is the chemical potential in the absence of coupling, and

(17) $$\Delta \mu \equiv \frac{\partial E_{\delta }}{\partial N}$$

is the additional chemical potential resulting from the energy exchange with the environment.

Only in systems where (6) is sufficiently close to 1, the interfacial energy contribution $E_{\delta }$ is negligible, and the internal energy E is extensive with respect to the system’s volume V and number of particles N:

$$\frac{E_{\delta }}{E}\to 0\;⟹\;\frac{\partial \left(\xi E\right)}{\partial \left(\xi V\right)}\to -p\;\&\;\frac{\partial \left(\xi E\right)}{\partial \left(\xi N\right)}\to \mu \;\;\forall \xi >0.$$

The nonextensivity of the energy E stems from the additional pressure (14), which increases the system’s energy by an amount

(18) $$E_{\delta ,p}=-V\Delta p,$$

and the additional chemical potential (17), which increases the energy with

(19) $$E_{\delta ,\mu }=N\Delta \mu .$$

The total interfacial energy $E_{\delta }$ in (3) is given by the sum of these two contributions:

(20) $$E_{\delta }=E_{\delta ,p}+E_{\delta ,\mu }.$$

While the analysis above was done for a system subject to the canonical constraints (β,N,V), a similar analysis may be carried out for other environmental variables by simply expanding the effective Boltzmann factor in (8). In the isothermal-isobaric (β,p,N) ensemble, the system may exchange work with its surroundings, and the Boltzmann factor is augmented with $e^{-pV_{j}}$, where *p* is the environmental pressure and $V_{j}$ are effective volume states. This results in a volume V given by (2) with $V\equiv \langle V_{j}\rangle$ and $V_{\delta }\equiv -T\langle \partial V_{j}/\partial T\rangle$. In this case, the nonextensive energy contribution $E_{\delta }$ is given by (19) alone.

In the grand canonical (β,μ,V) ensemble, the Boltzmann factor is corrected with $e^{\mu n}$, where μ is the chemical potential and *n* is the effective number of particles in the strongly coupled system. This results in a number of particles N given by (4) with $N\equiv \langle n\rangle$ and $N_{\delta }\equiv -T\langle \partial n/\partial T\rangle$. In this case, the nonextensive energy contribution is simply (18).

The nonextensive energy contribution in each of the three ensembles is different, making them nonequivalent. However, as the system becomes large with respect to the interaction region, expressions (18)–(20) all become zero, and, as expected, all ensemble descriptions are equivalent.

## 4. The Interaction Region

Traditional treatments of the system’s boundary regard it as an infinitely thin *dividing surface* [23] with excess variables [24]. As we illustrate in Figure 1, the framework of thermodynamics at strong coupling allows us to construct an effective interaction region containing not only $N_{\delta }$ particles and energy $E_{\delta }$, but also a volume $V_{\delta }$ that surrounds a bare system with *N* particles, energy *E*, and volume *V*.

From (2), (4), and (18)–(20), it follows that the interaction energy $E_{\delta }$ is given by

(21) $$E_{\delta }=-\left(V+V_{\delta }\right)\Delta p+\left(N+N_{\delta }\right)\Delta \mu .$$

Then, the pressure $p_{\delta }$ and chemical potential $\mu_{\delta }$ in the interaction region are, respectively, given by

(22) $$p_{\delta }=-\frac{\partial E_{\delta }}{\partial V_{\delta }}=\Delta p,$$

(23) $$\mu_{\delta }=\frac{\partial E_{\delta }}{\partial N_{\delta }}=\Delta \mu ,$$

where the second equalities stem from (14) and (17).

In contrast to classical thermodynamic theory, this framework considers the possibility of nonextensivity by describing the system always in conjunction with the interaction phase $\left(E_{\delta },V_{\delta },N_{\delta }\right)$ surrounding it. As is well known, while classical thermodynamics does have its range of applicability, it cannot be relied upon to describe the properties of nanoscale systems where perturbations from the surroundings are very significant. However, increasingly accurate experiments and simulations show that select expressions of classical thermodynamics are surprisingly accurate at describing the properties of nanosystems. In the following, we show that TSC’s treatment of the system’s interaction region naturally produces thermodynamic laws that are valid at the nanoscale.

### 4.1. Capillary Pressure

Despite its strictly classical origin (over two centuries ago), the Young–Laplace law was recently shown to accurately describe capillary pressure in nanopores as small as 1–2 nm [25]. As we shall see, this law’s adequacy to describe strongly coupled systems emerges as a simple result from the framework of TSC.

The capillary pressure between two static fluids separated by a curved surface is nothing but the pressure in the interaction region connecting both fluids. In contrast to a classical treatment of the interface as a geometrical surface, TSC treats the interaction region as a phase with volume $V_{\delta }$ and energy $E_{\delta }$. The pressure in this phase is given by (22), and it may be expressed as

(24) $$p_{\delta }=-\frac{\partial_{X}E_{\delta }}{\partial_{X}V_{\delta }},$$

for some variable *X*. If *X* is taken to be the area *A* of the bare system’s boundary, then (24) becomes

(25) $$p_{\delta }=-\gamma \frac{\partial A}{\partial V_{\delta }},$$

where

(26) $$\gamma \equiv \frac{\partial E_{\delta }}{\partial A}$$

is known as the *surface tension*. If the sum of the system’s radius *r* and the shell thickness δ is assumed constant, then the total volume V in (2) is constant, and (25) produces the Young-Laplace law:

(27) $$p_{\delta }=\gamma \frac{\partial A}{\partial V}=\gamma \frac{\partial_{r}A\left(r\right)}{\partial_{r}V\left(r\right)}=\frac{2}{r}\gamma ,$$

where the last equality applies to a variety of geometries, including spherical systems, toroidal droplets, and capillary tubes.

### 4.2. Capillary Condensation

Just like expression (27) is valid in the strong coupling regime described by TSC, recent experiments in capillary tubes with radii as small as 8 nm [26] have shown the nanoscale validity of Kelvin’s classical relation for vapor pressure and capillary condensation. In the framework of TSC, capillary condensation is governed by the chemical potential (23) in the interaction region, which, using (22) and (27), may be expressed in terms of the surface tension as

(28) $$\Delta \mu =-\frac{p_{\delta }}{\rho_{\delta }}=-\frac{2\gamma }{r\rho_{\delta }}$$

where $\rho_{\delta }$ is the density in the interaction phase, defined, like (22) and (23), as

(29) $$\rho_{\delta }\equiv \frac{\partial N_{\delta }}{\partial V_{\delta }}=\hat{\rho }-\rho ,$$

$\hat{\rho }$ is the density of the system, and ρ is the density of the surrounding bath. If we consider the system to be a vapor bubble with saturated vapor pressure $\hat{p}$ inside a liquid phase with vapor pressure *p*, then $\rho \gg \hat{\rho }$ and $\Delta \mu \approx k_{B}T\log\hat{p}/p$, which inserted in (28) and (29) results in the Kelvin equation

(30) $$p\approx \hat{p}\;\exp\left(-\frac{2\gamma /r\rho }{k_{B}T}\right).$$

This result is valid for bubbles and, as shown by recent nanoscale experiments [26], even in capillary tubes that are far too narrow for bubbles to form.

### 4.3. Wetting

**Figure 2:**
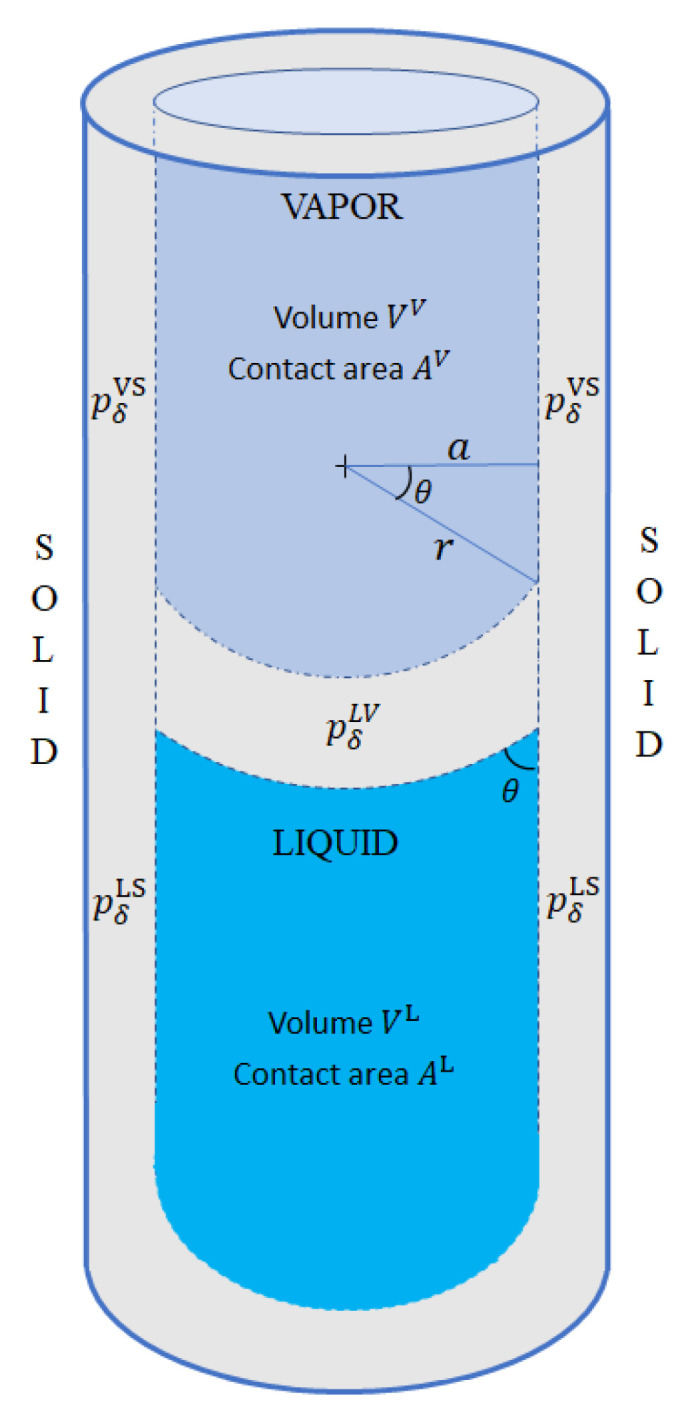
Capillary tube with three phases and three interfacial regions. The difference between $p_{\delta }^{\mathrm{VS}}$ and $p_{\delta }^{\mathrm{LS}}$ causes the wetting angle θ to deviate from 90°. This causes in turn an interfacial pressure $p_{\delta }^{\mathrm{LV}}=p_{\delta }^{\mathrm{VS}}-p_{\delta }^{\mathrm{LS}}$ between the liquid and the vapor phases.

Applying TSC in narrow capillary tubes demands that, in addition to the three phases (liquid, vapor, and solid), we also consider three interaction regions, namely, liquid–vapor (LV), liquid–solid (LS), and vapor–solid (VS). Each of the three interaction phases has its volume ($V_{\delta }^{\mathrm{LV}}$, $V_{\delta }^{\mathrm{LS}}$, $V_{\delta }^{\mathrm{VS}}$), energy ($E_{\delta }^{\mathrm{LV}}$, $E_{\delta }^{\mathrm{LS}}$, $E_{\delta }^{\mathrm{VS}}$), and its corresponding pressure

(31) $$p_{\delta }^{i}=-\frac{\partial E_{\delta }^{i}}{\partial V_{\delta }^{i}},$$

where i={LV,LS,VS}, as shown in Figure 2.

The curvature between the liquid and vapor phases is caused by an imbalance between $p_{\delta }^{\mathrm{VS}}$ and $p_{\delta }^{\mathrm{LS}}$. When $p_{\delta }^{\mathrm{VS}}>p_{\delta }^{\mathrm{LS}}$, more vapor than liquid is pushed away from the solid surface, and the wetting angle θ becomes acute. On the other hand, if $p_{\delta }^{\mathrm{VS}}<p_{\delta }^{\mathrm{LS}}$, the contact angle θ becomes obtuse. In either case, the resulting curvature creates a surface tension $\gamma^{\mathrm{LV}}$ with its corresponding pressure $p_{\delta }^{\mathrm{LV}}$ given by the Young–Laplace law (27)

(32) $$p_{\delta }^{\mathrm{LV}}=\frac{2}{r}\gamma^{\mathrm{LV}}=\frac{2}{a}\gamma^{\mathrm{LV}}\cos\theta ,$$

and by the imbalance between $p_{\delta }^{\mathrm{VS}}$ and $p_{\delta }^{\mathrm{LS}}$

(33) $$p_{\delta }^{\mathrm{LV}}=p_{\delta }^{\mathrm{VS}}-p_{\delta }^{\mathrm{LS}},$$

each of which is given by (27)

(34) $$p_{\delta }^{\mathrm{LS}}=\frac{\partial A^{L}}{\partial V^{L}}\gamma^{\mathrm{LS}}=\frac{2}{a}\gamma^{\mathrm{LS}},$$

(35) $$p_{\delta }^{\mathrm{VS}}=\frac{\partial A^{V}}{\partial V^{V}}\gamma^{\mathrm{VS}}=\frac{2}{a}\gamma^{\mathrm{LS}}.$$

Combining the last four expressions produces Young’s wetting equation:

(36) $$\gamma^{\mathrm{LV}}\cos\theta =\gamma^{\mathrm{VS}}-\gamma^{\mathrm{LS}}.$$

The accuracy of this old expression for describing strongly coupled nanosystems has been verified by experiments and simulations with carbon nanotubes and nanocones [27].

### 4.4. Tolman Length

How much does the surface tension γ between two curved phases deviate from the planar value $\gamma_{0}$? In 1949, Tolman used elaborate classical theory and proposed a law (see in [28] eq. 4.3) whose applicability at the nanoscale has just been established [29]. As shown below, this law, and its applicability to small, strongly coupled systems, becomes a straightforward result in the framework of TSC.

Considering an interaction phase of thickness δ around a spherical system with volume *V*, as illustrated in Figure 1, the right hand side of (25) becomes

(37) $$\frac{\partial V_{\delta }}{\partial A}=\frac{\partial_{V}V_{\delta }\left(V;\delta \right)}{\partial_{V}A\left(V\right)}=\;\delta \left(1+\frac{\delta }{r}+\frac{1}{3}\frac{\delta^{2}}{r^{2}}\right),$$

and (25) obeys

(38) $$\frac{d\gamma }{dp_{\delta }}=-\delta \left(1+\frac{\delta }{r}+\frac{1}{3}\frac{\delta^{2}}{r^{2}}\right).$$

Inserting (27) into (38) produces the the Gibbs–Tolman–Koening–Buff equation (see in [28] eq. 4.1):

(39) $$\frac{1}{\gamma }\frac{d\gamma }{dr}=\frac{2\frac{\delta }{r^{2}}\left(1+\frac{\delta }{r}+\frac{1}{3}\frac{\delta^{2}}{r^{2}}\right)}{1+2\frac{\delta }{r}\left(1+\frac{\delta }{r}+\frac{1}{3}\frac{\delta^{2}}{r^{2}}\right)},$$

which integrated from *∞* to *r* results in Tolman’s law

(40) $$\frac{\gamma }{\gamma_{0}}=1-2\frac{\delta }{r}+O{\left(\frac{\delta }{r}\right)}^{2},$$

where the quantity δ, i.e., the thickness of the interaction phase, is classically known as the *Tolman length*.

The examples above show that TSC’s framework naturally captures the properties of systems subject to strong coupling, smoothly predicting laws recently shown to be surprisingly accurate at the nanoscale. The method may be applied to predict the thermal properties of a wide range of nonextensive systems, such as macromolecules, nanoclusters, and quantum nanodevices.

## 5. Concluding Remarks

We have presented a theoretical framework capable of describing the thermodynamic properties of nonextensive systems by including the influence of the interaction phase surrounding the system. In contrast to theories based on a purely thermodynamic starting point, the Hamiltonian of mean force can account for the microscopic origin of nonextensivity and provide a general framework for nonextensive thermodynamics. Moreover, the Hamiltonian approach provides a better foundation for the modeling and simulation of complex systems regardless of their size.

A proper characterization of the interaction phase is indeed important to describe inherently nonextensive interfacial phenomena. While classical interfacial theory assumes an infinitely thin surface of discontinuity, thermodynamics at strong coupling directly accounts for the interaction region surrounding a system, and it can describe the properties of interphases as easily as those of the systems they surround. This descriptive accessibility of the interfacial region is increasingly important as the scientific community becomes interested in ever smaller nanobiosystems whose properties are strongly influenced by the immediate environment [30,31], which in turn becomes of paramount importance for the system’s applications, design, and operation.
